# miR-142-3p Contributes to Early Cardiac Fate Decision of Embryonic Stem Cells

**DOI:** 10.1155/2017/1769298

**Published:** 2017-06-05

**Authors:** Zhong-Yan Chen, Fei Chen, Nan Cao, Zhi-Wen Zhou, Huang-Tian Yang

**Affiliations:** ^1^Key Laboratory of Stem Cell Biology and Laboratory of Molecular Cardiology, Institute of Health Sciences, Shanghai Jiao Tong University School of Medicine (SJTUSM) and Shanghai Institutes for Biological Sciences (SIBS), Chinese Academy of Sciences (CAS), Shanghai, China; ^2^Department of Cardiology, Shanghai Xuhui District Central Hospital, Shanghai, China

## Abstract

MicroRNAs (miRNAs) play important roles in cell fate decisions. However, the miRNAs and their targets involved in the regulation of cardiac lineage specification are largely unexplored. Here, we report novel functions of miR-142-3p in the regulation of cardiomyocyte differentiation from mouse embryonic stem cells (mESCs). With a miRNA array screen, we identified a number of miRNAs significantly changed during mESC differentiation into the mesodermal and cardiac progenitor cells, and miR-142-3p was one among the markedly downregulated miRNAs. Ectopic expression and inhibition of miR-142-3p did not alter the characteristics of undifferentiated ESCs, whereas ectopic expression of miR-142-3p impaired cardiomyocyte formation. In addition, ectopic expression of miR-142-3p inhibited the expression of a cardiac mesodermal marker gene *Mesp1* and downstream cardiac transcription factors *Nkx2.5*, *Tbx5*, and *Mef2c* but not the expression of three germ layer-specific genes. We further demonstrated that miR-142-3p targeted the 3′-untranslated region of *Mef2c*. These results reveal miR-142-3p as an important regulator of early cardiomyocyte differentiation. Our findings provide new knowledge for further understanding of roles and mechanisms of miRNAs as critical regulators of cardiomyocyte differentiation.

## 1. Introduction

Embryonic stem cells (ESCs), derived from the inner cell mass of blastocysts, are pluripotent and self-renewing cells with the ability to give rise to all derivatives of three germ layers [1]. Differentiation of ESCs mimics the early stage of embryonic development, including the cardiomyogenic lineage commitment, thus making ESCs an ideal model to study the regulators and mechanisms of in vivo mammalian development [2, 3]. Proper differentiation requires precise regulation of signalling pathways, epigenetic modification, and transcription networks [4–6]. Accumulating evidence has shown that posttranscriptional and posttranslational regulations of lineage-specific genes by small noncoding RNAs, such as microRNAs (miRNAs), play important roles in cell fate and lineage commitment of ESCs [7]. However, the specific miRNAs that control ESC differentiation have not yet been fully clarified.

miRNAs are 22- to 25-nucleotide-long, endogenous single-stranded noncoding RNAs that regulate gene expression at the posttranscriptional level by mRNA degradation or translation repression [8]. miRNAs play important roles in embryo development and cell fate decision, proliferation, and differentiation [9–11]. ESC-derived cardiomyocyte formation involves the formation of mesodermal cells, specification of mesodermal cells to cardiac progenitor cells (CPCs), and differentiation of CPCs into immature cardiomyocytes [4]. A number of miRNAs have been identified to regulate these stages. miR-1 and miR-133 promote mesoderm formation from ESCs but have opposing functions during further differentiation into CPCs [9]. miR-499 promotes differentiation of CPCs into cardiomyocytes [12]. We previously found that miR-125b/Lin28 axis is a critical regulator in the control of mesendodermal specification from mouse ESCs (mESCs) and subsequent cardiac differentiation [13]. However, whether there are other miRNAs that are involved in early cardiac differentiation needs to be further determined.

miR-142-3p, an evolutionally conserved miRNA of vertebrates, is a hematopoietic-specific miRNA [14] and regulates cell fate decision in the vertebrate hematopoietic system [15–17]. miR-142-3p is also a multifaceted regulator in organogenesis, homeostasis, and tumorigenesis [18]. Recently, miR-142-3p is reported to balance self-renewal and differentiation in mESCs via regulating KRAS/ERK signalling [19]. In addition, miR-142-3p is reported to regulate heart formation in zebrafish [20]. However, it is unknown whether miR-142-3p regulates mammalian cardiogenesis.

In this study, we screened miRNAs that might be involved in the differentiation of mESCs into mesoderm and CPCs by miRNA microarray and identified the changes of miR-142-3p abundance during mesodermal and early cardiac differentiation. We then examined the function of miR-142-3p on ESC self-renewal and cardiac differentiation and identified the potential targets. Our data showed that miR-142-3p is an important regulator for early cardiac differentiation of ESCs. These findings provide insights into the novel role of miR-142-3p in the regulation of cardiac lineage commitment and add information for the further development of cell therapy and drug discovery.

## 2. Materials and Methods

### 2.1. Culture and In Vitro Differentiation of ESCs

R1 and E14 mESCs carrying a Brachyury-GFP (T-GFP) were maintained on mitomycin C-inactivated mouse embryonic fibroblast cells as described previously [5, 21]. Cardiomyocyte differentiation was initiated by a hanging-drop technique [22]. In brief, ESCs were trypsinized and cultivated as embryoid bodies (EBs) in the absence of leukemia inhibitory factor (Millipore) for 2 days followed by 3 days of suspension cultured in the medium containing 10% FBS (Gibco, USA). Then, EBs were plated onto gelatin-coated tissue culture dishes. Differentiated cardiomyocytes appeared in the form of spontaneously contracting cell clusters. All cultivation medium and other reagents for cell culture were from Invitrogen (Carlsbad, CA, USA) unless indicated otherwise. E14 mESCs carrying a T-GFP were used for collecting mesodermal cells; R1 mESCs were used for other experiments.

### 2.2. Analysis of miRNA Expression Profiling

T-GFP^+^ mesodermal cells were isolated from day 3 EBs of E14 mESCs carrying T-GFP by fluorescence-activated cell sorting (FACS). FLK1^+^/CXCR4^+^ CPCs [23] were isolated from day 5 EBs of R1 mESCs by FACS. Total RNA was isolated with TRIzol (Invitrogen, USA). miRNA expression profiling was carried out with Agilent 8x60K mouse miRNA one-color microarray (V16.0) (Agilent Technologies Inc., Santa Clara, CA, USA). miRNA hybridization and data collection were conducted following the manufacturer's instructions.

### 2.3. Plasmid Construction and Cell Transfection

To generate miR-142-3p-overexpressing ESC lines, the miR-142 stem-loop flanked by 170 nucleotides on each side was amplified by polymerase chain reaction (PCR) from mouse genomic DNA and inserted into pCDH-EF1-MCS-T2A-Puro lentiviral vector. Then, the vector was transfected with pMD-VSVG, pRSV-REV, and pMDLG-PRRL into HEK293FT cells to generate lentivirus. ESCs were infected with the lentivirus, and then puro-resistant clones were picked 3 days after puromycin (Gibco/BRL, USA) selection and propagated. To construct the luciferase reporter plasmid, 3′UTR regions of the gene of interest were amplified by PCR from cDNA and inserted into a luciferase reporter vector psiCHECK-2. The mutated 3′UTR were generated by PCR-based site-directed mutagenesis. For the inhibition of miR-142-3p, ESCs were transfected with the commercialized miR-142-3p inhibitors or scramble (RiboBio, China) using Lipofectamine 2000 (Invitrogen, USA) according to the manufacturer's instructions.

### 2.4. Reverse Transcription PCR

Cells were collected in TRIzol (Invitrogen, USA) for total RNA isolation. 1 *μ*g of total RNA was reversed transcribed using oligo (dT) primer and ReverTra Ace reverse transcriptase (Toyobo, Japan). PCR was carried out using Taq PCR mix (Vazyme, China). The 28S ribosomal RNA was used for internal normalization. The primers used are listed in Supplementary Table S1 available online at https://doi.org/10.1155/2017/1769298.

### 2.5. Quantitative Real-Time PCR (qRT-PCR)

qRT-PCR was performed on an ABI 7900HT instrument (Applied Biosystems, USA) with SYBR Green Real-time PCR Master Mix (Toyobo, Japan). For mRNA detection, glyceraldehyde 3-phosphate dehydrogenase (*Gapdh*) was used for internal normalization. The primers used for mRNA detection are listed in Supplementary Table S1. For miRNA detection, reverse transcription and miRNA detection were carried out using the miRNA Reverse Transcription kit and TaqMan miRNA Expression Assays (Applied Biosystems, USA). Small nuclear RNA U6 was used for internal normalization.

### 2.6. Flow Cytometry Analysis

Undifferentiated ESCs or EBs were harvested and dissociated with trypsin. To detect SSEA1, samples were fixed with 1% paraformaldehyde, then stained for PE-conjugated SSEA1 antibody (1 : 20, eBioscience, USA) or isotype-matched negative control. To determine TNNT2^+^ cardiomyocytes, cells were fixed and permeabilized by Cytofix/Cytoperm™ Kit (BD Biosciences, USA), blocked by 5% FBS and incubated with primary antibody of TNNT2 (1 : 200, Abcam, UK) or isotype-matched IgG control. DyLight 549-conjugated antibodies (Jackson Lab, USA) were used as the secondary antibody. Cells were then analysed and quantified by flow cytometry (FACSAria, BD Biosciences, USA). For cell sorting, live cells were harvested and double-stained with APC-conjugated FLK1 (1 : 100, BD Biosciences, USA) and PE-conjugated CXCR4 (1 : 50, BD Biosciences, USA).

### 2.7. Immunocytochemical Staining

Alkaline phosphatase (ALP) activity was analysed by using an ALP substrate kit III (Vector Laboratories, USA) according to the manufacturer's instructions. Immunostaining assays were performed according to the protocol described previously [24]. Briefly, cells were fixed with 4% paraformaldehyde, permeabilized in 0.3% Triton X-100, blocked in 10% normal goat serum (Vector Laboratories), and then incubated with primary antibodies against OCT4 (1 : 200, Abcam, USA), NANOG (1 : 200, Abcam, USA), and TNNT2 (1 : 500; Abcam, USA) in 4°C overnight and detected by DyLight 488- or DyLight 549-conjugated secondary antibodies. Nuclei were stained with DAPI (Sigma, USA). A Zeiss Axio Observer A1 fluorescence microscope was used for slide observing and image capture.

### 2.8. Luciferase Reporter Assay

HEK293FT cells were cultured to 70% confluence in 24-well plates, and then transfected with a mixture of 100 ng of 3′UTR luciferase reporter plasmid and 50 nM miRNA mimics (RiboBio, China) in each well by Lipofectamine 2000 (Invitrogen, USA). Cell lysates were harvested 24 h after transfection, and reporter activity was measured with the Dual Luciferase Assay kit (Promega, USA) according to the manufacturer's instruction.

### 2.9. Bioinformatics Analysis

RNAhybrid and miRanda were used to predict potential targets.

### 2.10. Statistical Analysis

Data were expressed as mean ± SEM. Statistical significance of differences was estimated by one-way ANOVA and two-tailed unpaired Student's *t*-test by GraphPad Prism 6.0. *P* < 0.05 was considered statistically significant.

## 3. Results

### 3.1. Expression Profiles of miRNAs during Early Cardiac Differentiation Stage

**Figure 1:**
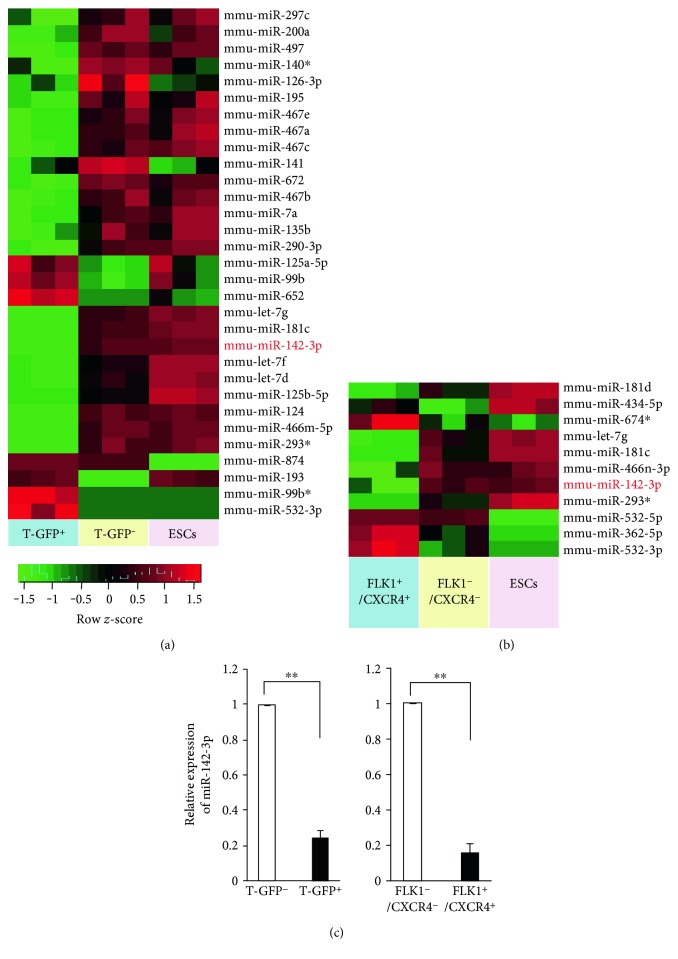
Microarray assay reveals miR-142-3p as the most downregulated miRNA. (a) Heat map representing hierarchical clustering of all miRNAs that displayed a 2-fold or greater difference in T-GFP^+^ cells compared to T-GFP^−^ cells. (b) Heat map representing hierarchical clustering of all miRNAs that displayed a 2-fold or greater difference in FLK1^+^/CXCR4^+^ cells compared to FLK1^−^/CXCR4^−^ cells. (c) Validation of miR-142-3p expression in T-GFP^+^ mesodermal cells and FLK1^+^/CXCR4^+^ CPCs by qRT-PCR. *n* = 3, ^∗∗^*P* < 0.01.

To identify miRNAs that might be involved in the cardiac lineage commitment, we screened miRNA expression profiles at the mesodermal and cardiac progenitor stages, which are essential for the cardiac lineage commitment [25]. mESC-derived T-GFP^+^ mesodermal cells [26] at differentiation day 3 and FLK1^+^/CXCR4^+^ CPCs [23] at differentiation day 5 were isolated from corresponding T-GFP^−^ and FLK1^−^/CXCR4^−^ populations by FACS (Supplemental Figure S1A–C) as previously described [27]. The enriched T-GFP^+^ and FLK1^+^/CXCR4^+^ fractions were confirmed by RT-PCR analysis of mesodermal marker *T* and cardiac progenitor marker *Nkx2.5*, *Isl1*, *Tbx5*, and *Mef2c*. (Supplemental Figure S1D-E). Then, miRNA microarray was used to compare miRNA expression profiles between T-GFP^+^ mesodermal cells and T-GFP^−^ cells as well as between FLK1^+^/CXCR4^+^ cells and FLK1^−^/CXCR4^−^ cells. 29 miRNAs showed more than 2-fold change in T-GFP^+^ mesoderm cells compared with the T-GFP^−^ cells (Table 1). 11 miRNAs showed more than 2-fold change in FLK1^+^/CXCR4^+^ CPCs compared with FLK1^−^/CXCR4^−^ cells (Table 2). The heat map image of hierarchical cluster of those miRNAs revealed a distinguished grouping of miRNA expression patterns (Figures 1(a) and 1(b)). Among these miRNAs, miR-142-3p was downregulated in both T-enriched mesoderm and FLK1^+^/CXCR4^+^ CPCs. This was further confirmed by qRT-PCR analysis (Figure 1(c)).

### 3.2. Ectopic Expression of miR-142-3p Does Not Affect Self-Renewal of ESCs

**Figure 2:**
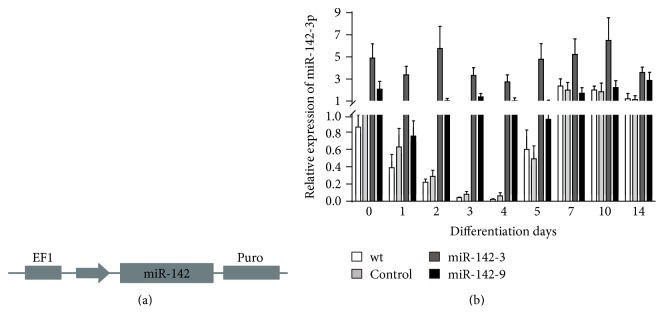
Establishment of miR-142-3p overexpression ESC lines. (a) Diagram depicting the construction of the miR-142-3p overexpression plasmid. (b) qRT-PCR analysis for the expression of miR-142-3p among wild-type (wt), blank vector control (control), and miR-142-3p overexpression ESC lines (miR-142-3 and miR-142-9, lower panel). *n* = 5.

**Figure 3:**
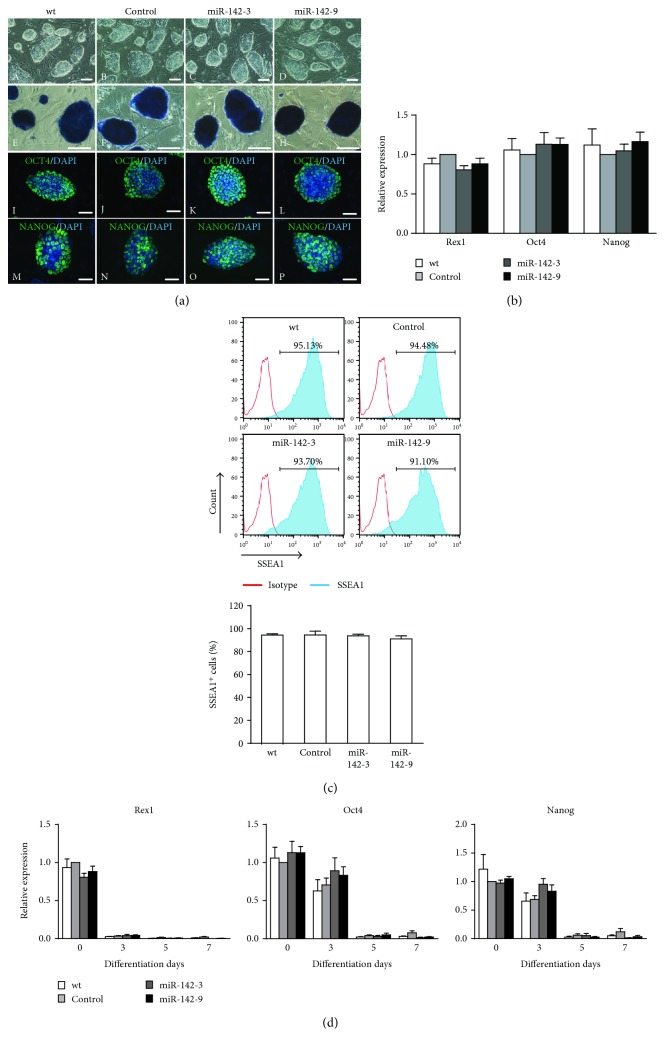
miR-142-3p overexpression does not affect the self-renewal of ESCs. (a) Morphology of the colonies of ESCs. (A–D) Phase-contrast images show undifferentiated ESC colonies. (E–H) ALP staining of ESC colonies. Immunostaining analysis of OCT4 (I–L) and NANOG (M–P). Scale bar: A–D = 100 *μ*m, E–P = 50 *μ*m. (b) qRT-PCR analysis for the expression of the pluripotency genes (*n* = 3). (c) Flow cytometry analysis of SSEA1 (*n* = 3). (d) qRT-PCR analysis for the expression of pluripotency genes during differentiation (*n* = 3).

To determine the role of miR-142-3p in self-renewal and differentiation, we established ESC lines stably expressing miR-142-3p by using lentivirus (Figure 2(a)). As shown in Figure 2(b), in wild-type (wt) cells, miR-142-3p was highly expressed in undifferentiated ESCs and declined at differentiation day 1, reaching nadir at differentiation day 4 and then gradually returned to baseline. The expression pattern of miR-142-3p in control cells transfected with the blank vector was comparable with the wt cells either in undifferentiated ESCs or in differentiating ESCs (Figure 2(b)). In undifferentiated status, the expression level of miR-142-3p in the overexpression cell lines (clones miR-142-3 and miR-142-9) was about 2- to 5-fold higher than those in wt and control cells (Figure 2(b)). During differentiation, the expression level of miR-142-3p in the overexpression cell lines remain the same as in undifferentiated status (Figure 2(b)). To confirm whether ectopic expression of miR-142-3p would affect the self-renewal property of ESCs, we compared characteristics of undifferentiated wt, control, and miR-142-3p overexpression ESCs. No significant differences in the colony morphology (Figure 3(a), A–D), ALP activity (Figure 3(a), E–H), and protein expression of pluripotency markers OCT4 and NANOG (Figure 3(a), I–P) were observed among these groups. qRT-PCR analysis also showed comparable level of *Rex1*, *Oct4*, and *Nanog* (Figure 3(b)). Flow cytometry analysis further confirmed that the number of cells expressing stage-specific embryonic antigen 1 (SSEA1) was similar among those cells (Figure 3(c)). The effect of miR-142 overexpression on the self-renewal of ESCs during differentiation was further examined, and there were no significant changes in the expression levels of pluripotency marker *Rex1*, *Oct4*, and *Nanog* in miR-142 overexpression cells compared with wt and control cells during differentiation (Figure 3(d)). To further determine the role of miR-142-3p in the self-renewal of ESCs, we suppressed the expression of miR-142-3p by using commercialized inhibitor (Supplementary Figure S2A). The cells transfected with scramble or miR-142-3p inhibitor showed similar levels of ALP activity (Supplementary Figure S2B, a-b), protein expression of pluripotency markers OCT4 and NANOG (Supplementary Figure S2B, c–f), pluripotency marker genes *Oct4*, *Nanog*, and *Rex1* (Supplementary Figure S2C), and the percentage of SSEA1^+^ cells (Supplementary Figure S2D). Taken together, these data indicate that miR-142-3p appears to be dispensable for maintaining self-renewal of ESCs.

### 3.3. miR-142-3p Suppresses Cardiomyocyte Differentiation

**Figure 4:**
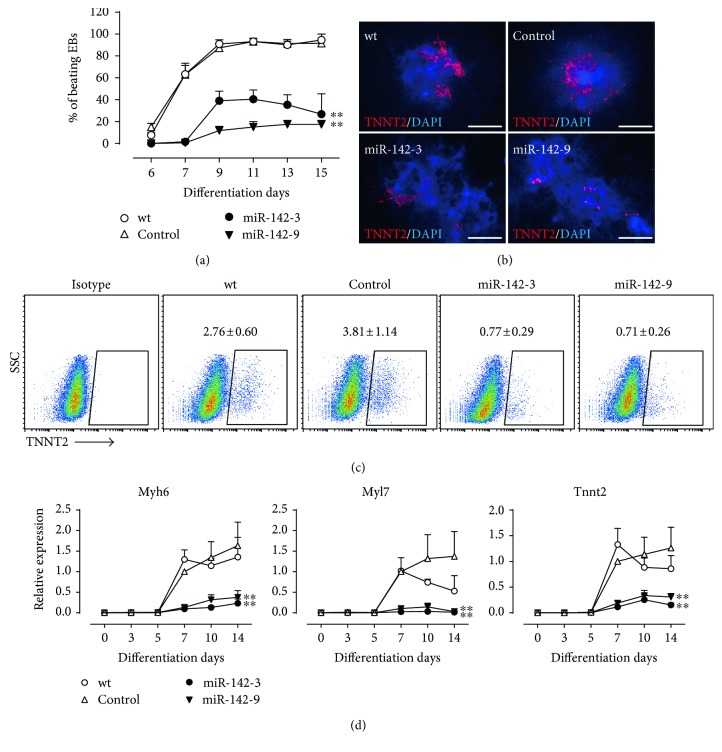
miR-142-3p overexpression suppresses cardiomyocyte differentiation of ESCs. (a) The percentage of EBs with spontaneously contracting cardiomyocytes during EB differentiation (*n* = 3). (b) Immunostaining analysis of TNNT2 in day 10 EBs. Scale bar = 500 *μ*m. (c) Flow cytometry analysis of TNNT2 in day 10 EBs (*n* = 3). (d) qRT-PCR analysis for the expression of the cardiac myofilament genes (*n* = 3). ^∗∗^*P* < 0.01 versus control.

To investigate the role of miR-142-3p during cardiac lineage commitment, ESCs were differentiated into cardiomyocytes by the EB formation. In wt and control ESCs, spontaneously contracting cardiomyocytes were visible at day 6, and the percentage of EBs containing spontaneously contracting cardiomyocytes increased gradually over time and reached over 90%, while in miR-142-3p overexpression ESCs, it dropped to 20% to 35% (Figure 4(a)). However, the number of EBs containing spontaneously contracting cardiomyocytes was indistinguishable between scramble and miR-142-3p-knockdown cells (Supplemental Figure S2E). Immunofluorescence staining confirmed that the positive area of cardiac myofilament protein TNNT2 was significantly smaller in miR-142-3p overexpression EBs than that in wt and control (Figure 4(b)). Flow cytometry analysis further confirmed that miR-142-3p overexpression decreased the percentage of TNNT2^+^ cardiomyocytes at differentiation day 10 (Figure 4(c)). Moreover, qRT-PCR analysis showed that the expression levels of cardiac myofilament genes *Myh6*, *Myl7*, and *Tnnt2* were markedly suppressed by miR-142-3p overexpression (Figure 4(d)). These data indicate that miR-142-3p negatively regulates cardiac differentiation.

### 3.4. miR-142-3p Suppresses ESC Differentiation into CPCs but Not Mesoderm Formation

**Figure 5:**
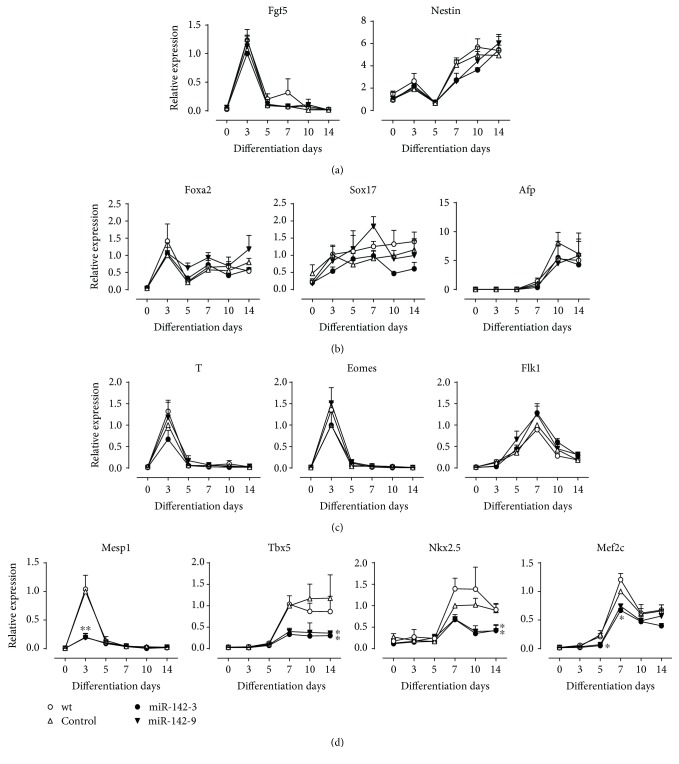
qRT-PCR analysis of differentiation marker genes during ESC differentiation. (a) Expression of ectodermal markers. (b) Expression of endodermal markers. (c) Expression of mesodermal markers. (d) Expression of cardiac progenitor markers (*n* = 6). ^∗^*P* < 0.05 versus control, ^∗∗^*P* < 0.01 versus control.

In vitro cardiomyocyte differentiation involves the specification of pluripotent cells to mesoderm and cardiac progenitors prior to terminal differentiation. To elucidate which differentiation stage is affected by miR-142-3p, we analysed the expression of germ layer and cardiac precursor genes by qRT-PCR. miR-142-3p overexpression did not significantly affect the expression of ectodermal (*Fgf5*, *Nestin*), endodermal (*Fox2*, *Sox17*, and *Afp*), and mesodermal (*T*, *Eomes*, and *Flk1*) marker genes (Figures 5(a), 5(b), and 5(c)). However, the expression of cardiac mesodermal gene *Mesp1* and cardiac progenitor genes *Tbx5*, *Nkx2.5*, and *Mef2c* were remarkably decreased (Figure 5(d)). Taken together, these data suggest that miR-142-3p decreases the populations of cardiac mesoderm and progenitor cells but not mesoderm formation of ESCs.

### 3.5. miR-142-3p Targets Mef2c in Cardiac Differentiation of ESCs

**Figure 6:**
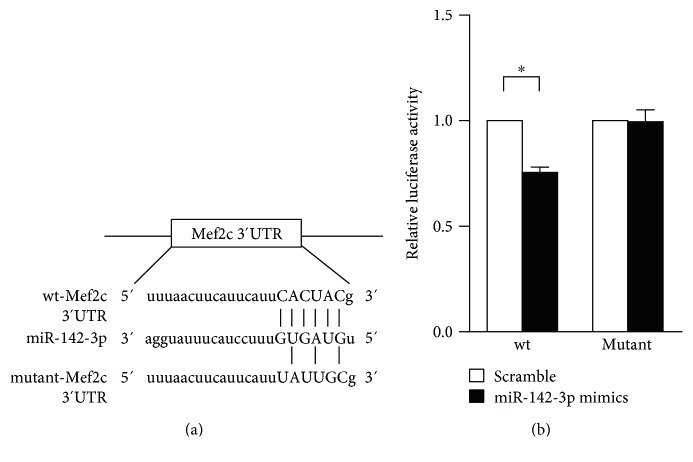
*Mef2c* is a downstream target of miR-142-3p. (a) The sketch construction of the wt and mutant *Mef2c* 3′UTR. (b) Relative luciferase activity of wt and mutant *Mef2c* 3′UTR vectors transfected with scramble control or miR-142-3p mimics (*n* = 5). ^∗^*P* < 0.05.

To elucidate the mechanisms by which miR-142-3p regulates cardiac differentiation, we searched for potential targets of miR-142-3p by using miRanda [28] and RNAhybrid [29]. Since *Mesp1* is the earliest marker of cardiovascular development [30], we examined whether miR-142-3p directly targets *Mesp1*. miR-142-3p was predicted to bind to the 3′UTR of *Mesp1* (Supplemental Figure S3A). However, when the 3′UTR of *Mesp1* was cloned into the luciferase reporter, miR-142-3p had no effect on the luciferase activity (Supplemental Figure S3B), indicating that miR-142-3p does not directly target *Mesp1*. Further analysis showed that the 3′UTR of *Mef2c*, a key regulator of cardiomyocyte formation [31], had a miR-142-3p binding site (Figure 6(a)). We then cloned the full length of the wt and mutant 3′UTR of mouse *Mef2c* into the downstream of the luciferase reporter. miR-142-3p reduced the activity of the luciferase reporter bearing wt 3′UTR of *Mef2c*. By contrast, miR-142-3p did not affect the activity of the luciferase reporter bearing mutant 3′UTR of *Mef2c* (Figure 6(b)). Moreover, qRT-PCR analysis showed that the expression of *Mef2c* was decreased by miR-142-3p (Figure 4(d)). Taken together, these results suggest that *Mef2c* may be the target of miR-142-3p.

## 4. Discussion

Here, we showed that (i) a number of miRNAs are significantly changed during the differentiation of mesodermal and cardiac progenitor cells from ESCs; (ii) miR-142-3p is highly expressed in undifferentiated ESCs, while it is downregulated during early ESC differentiation and its expression is significantly lower in T-GFP^+^ cells and FLK1^+^/CXCR4^+^ CPCs than in corresponding T-GFP^−^ cells and FLK1^−^/CXCR4^−^ cells; (iii) ectopic expression of miR-142-3p does not affect the self-renewal and germ layer specification of ESCs, whereas it suppresses cardiomyocyte formation; (iv) this inhibition is associated with the downregulation of the expression of cardiac mesodermal marker gene *Mesp1* and the downstream cardiac progenitor marker genes *Nkx2.5*, *Tbx5*, and *Mef2c*; and (v) miR-142-3p targets the 3′UTR of *Mef2c*. These findings reveal a novel role of miR-142-3p in the regulation of cardiac lineage fate decision and provide its potential mechanism underlying the control of cell lineage decision and cardiogenesis.

Our results show that both gain and loss of function of miR-142-3p do not affect the self-renewal of undifferentiated ESCs. This is consistent with the recent report that in undifferentiated ESCs, there are high miR-142 and low miR-142 populations, while the two populations are indistinguishable by pluripotency markers [19]. They also reported that constitutive expression of miR-142 locks ESCs in an undifferentiated state when exposed to differentiation cues [19]. However, we did not observe a significant delay of decrease of pluripotency genes upon differentiation in miR-142-3p overexpression cells. Such conflicted findings may be caused by the different overexpression levels in undifferentiated state. It may also be caused by the difference in differentiation models used. Sladitschek and Neveu [19] induced ESC differentiation by using various cytokines, while we used the EB model without addition of any cytokines.

During the cardiomyocyte differentiation from ESCs, the cardiac mesoderm and CPC formation is critical to the cardiac lineage fate decision [25]. *Mesp1* is the master regulator of cardiac lineage commitment and is the earliest marker of cardiovascular development [30, 32]. It is transiently expressed in the nascent mesoderm, and it specifies mesodermal cells toward cardiac lineage by triggering the expression of cardiac markers [32]. Our data showed that miR-142-3p negatively regulates the formation of cardiac mesoderm and CPCs, and the subsequent cardiomyocyte differentiation. Downregulation of miR-142-3p during ESC differentiation is required for the specification of mesodermal cells to CPCs. This is supported by the findings that (i) miR-142-3p is downregulated in mesodermal and CPC populations; (ii) miR-142-3p does not affect the formation of nascent mesoderm; (iii) miR-142-3p inhibits the expression of *Mesp1* and the downstream cardiac progenitor genes; and (iv) miR-142-3p directly targets *Mef2c*, though further validation at the protein level is needed. Notably, no further increase in the cardiac differentiation is observed by knockdown of miR-142-3p, suggesting that the endogenous decline of miR-142-3p reaches the saturation level to allow sufficient CPC differentiation and subsequent cardiomyocyte formation. We found that miR-142-3p may function upstream of *Mesp1* through an indirect regulatory way. The mechanism by which miR-142-3p regulates *Mesp1* needs further investigation.

Interestingly, the role of miR-142-3p in cardiac lineage commitment seems different between zebrafish and mESCs. Knockdown of miR-142-3p during the development of zebrafish disrupts normal cardiac formation and function [20], while in the present study, miR-142-3p overexpression suppresses cardiomyocyte formation. Such difference suggests a species-dependent role of miR-142-3p. The in vivo role of miR-142-3p on cardiac development among various mammalian systems requires further investigation.

In the microarray results, some miRNAs showed similar expression pattern to that of miR-142-3p, such as miR-125b-5p, miR-124, and let-7g. We previously found that miR-125b-5p is downregulated during differentiation and controls cardiac differentiation via regulating mesendodermal specification [13]. miR-124 is a neuron-specific miRNA and known to be involved in neurogenesis [33]. Recently, it has been reported to regulate cardiomyocyte differentiation of bone marrow-derived mesenchymal stem cells [34]. However, whether miR-124 regulates cardiomyocyte differentiation remains unknown. The let-7 family member let-7c is involved in the control of cardiomyocyte differentiation by directly targeting the polycomb complex group protein *Ezh2* [35], but it is unclear whether let-7g regulates cardiomyocyte differentiation. It needs to be determined whether these miRNAs might work together with miR-142-3p in the regulation of cardiac differentiation of ESCs.

In summary, our results reveal a number of miRNAs potentially involved in ESC differentiation into mesodermal and cardiac progenitor cells. miR-142-3p negatively regulates the differentiation of cardiomyocyte through affecting the specification of cardiac mesodermal cells and CPCs.

## Supplementary Material

Table S1. List of primer sequences for PCR detection. Figure S1. Isolation and characterization of the samples for microRNA microarray assay. (A) Schematic diagram of the strategy for sample collection. (B) FACS sorting of T-GFP- and T-GFP+ subpopulations and the purity detection. (C) FACS sorting of FLK1-/CXCR4- and FLK1+/CXCR4+ subpopulations and the purity detection. (D) RT-PCR analysis of T expression in unsorted, T-GFP- and T-GFP+ cells. (E) RT-PCR analysis of cardiac progenitor marker genes in unsorted, FLK1- /CXCR4- and FLK1+/CXCR4+ subpopulations. Figure S2. Knockdown of miR-142-3p does not affect the self-renewal and cardiomyocyte differentiation of ESCs. ESCs were transfected with 100 nM miR-142-3p inhibitor or scramble control for 48h. (A) qRT-PCR analysis of miR-142-3p in ESCs transfection with 100 nM miR-142-3p inhibitor or scramble control. (B) ALP staining of the colonies of ESCs (a-b). Immunostaining analysis of OCT4 (c-d) and NANOG (e-f). scale bar: a-b =100 mm,c-f =50 mm. (C) qRT-PCR analysis for the expression of the pluripotency marker genes. n=3. (D) Flow cytometry analysis of SSEA1. n=3. (E) The percentage of EBs with contracting clusters during differentiation. n=3. Figure S3. miR-142-3p does not directly target to the 3′UTR of Mesp1. (A) RNAHybrid predicts the binding of miR-142-3p to the 3′UTR of Mesp1. (B) Luciferase assay determined in HEK293T cells that were transfected with the 3′UTR reporter construct together with miR-142-3p mimics or scramble. n=3.

## Figures and Tables

**Table 1:** Fold change of the miRNAs in T-GFP^+^ versus T-GFP^−^.

miRNA ID	Fold change	*P* value
mmu-miR-193	134.10	0.000613
mmu-miR-99b^∗^	85.31	0.002295
mmu-miR-532-3p	44.47	0.017969
mmu-miR-99b	2.85	0.008900
mmu-miR-652	2.36	0.003940
mmu-miR-125a-5p	2.20	0.016116
mmu-miR-874	2.07	0.004076
mmu-miR-140^∗^	−2.01	0.036421
mmu-miR-467c	−2.15	0.000925
mmu-miR-467a	−2.23	0.031586
mmu-miR-141	−2.24	0.045208
mmu-miR-297c	−2.32	0.015306
mmu-miR-135b	−2.33	0.020035
mmu-miR-497	−2.34	0.000315
mmu-miR-290-3p	−2.34	0.001229
mmu-miR-467b	−2.57	0.004658
mmu-miR-126-3p	−2.59	0.025740
mmu-miR-200a	−2.63	0.001876
mmu-miR-467e	−2.71	0.011901
mmu-miR-195	−2.73	0.006600
mmu-miR-7a	−3.04	0.012637
mmu-miR-672	−14.85	0.002030
mmu-miR-466m-5p	−76.86	0.004111
mmu-let-7f	−85.32	0.001921
mmu-miR-181c	−92.73	0.000002
mmu-miR-125b-5p	−99.27	0.000697
mmu-miR-124	−105.00	0.000622
mmu-let-7g	−115.25	0.000002
mmu-miR-142-3p	**−214.59**	**0.001338**

^∗^The element of the miRNA names based on the miRNA nomenclature.

**Table 2:** Fold change of the miRNAs in FLK1^+^/CXCR4^+^ versus FLK1^−^/CXCR4^−^.

miRNA ID	Fold change	*P* value
mmu-miR-362-5p	8.73	0.0201
mmu-miR-532-3p	7.16	0.0293
mmu-miR-674^∗^	2.48	0.0257
mmu-miR-434-5p	2.39	0.0126
mmu-miR-532-5p	2.07	0.0027
mmu-miR-181d	−2.58	0.0193
mmu-miR-293^∗^	−5.98	0.0359
mmu-miR-466n-3p	−20.89	0.0345
mmu-miR-142-3p	**−46.98**	**0.0308**
mmu-miR-181c	−57.34	0.0454
mmu-let-7g	−83.97	0.0240

^∗^The element of the miRNA names based on the miRNA nomenclature.
