# Supplementary material for: miR-142-3p Contributes to Early Cardiac Fate Decision of Embryonic Stem Cells
Source: Stem Cells Int. 2017 Jun 5;2017:1769298. doi: 10.1155/2017/1769298 (PMC5474537; doi:10.1155/2017/1769298)
Supplement: Supplementary file 1 — Table S1. List of primer sequences for PCR detection. Figure S1. Isolation and characterization of the samples for microRNA microarray assay. (A) Schematic diagram of the strategy for sample collection. (B) FACS sorting of T-GFP- and T-GFP+ subpopulations and the purity detection. (C) FACS sorting of FLK1-/CXCR4- and FLK1+/CXCR4+ subpopulations and the purity detection. (D) RT-PCR analysis of T expression in unsorted, T-GFP- and T-GFP+ cells. (E) RT-PCR analysis of cardiac progenitor marker genes in unsorted, FLK1- /CXCR4- and FLK1+/CXCR4+ subpopulations. Figure S2. Knockdown of miR-142-3p does not affect the self-renewal and cardiomyocyte differentiation of ESCs. ESCs were transfected with 100 nM miR-142-3p inhibitor or scramble control for 48h. (A) qRT-PCR analysis of miR-142-3p in ESCs transfection with 100 nM miR-142-3p inhibitor or scramble control. (B) ALP staining of the colonies of ESCs (a-b). Immunostaining analysis of OCT4 (c-d) and NANOG (e-f). scale bar: a-b =100 mm,c-f =50 mm. (C) qRT-PCR analysis for the expression of the pluripotency marker genes. n=3. (D) Flow cytometry analysis of SSEA1. n=3. (E) The percentage of EBs with contracting clusters during differentiation. n=3. Figure S3. miR-142-3p does not directly target to the 3′UTR of Mesp1. (A) RNAHybrid predicts the binding of miR-142-3p to the 3′UTR of Mesp1. (B) Luciferase assay determined in HEK293T cells that were transfected with the 3′UTR reporter construct together with miR-142-3p mimics or scramble. n=3. [file 1769298.f1.pptx]

## Slide 1
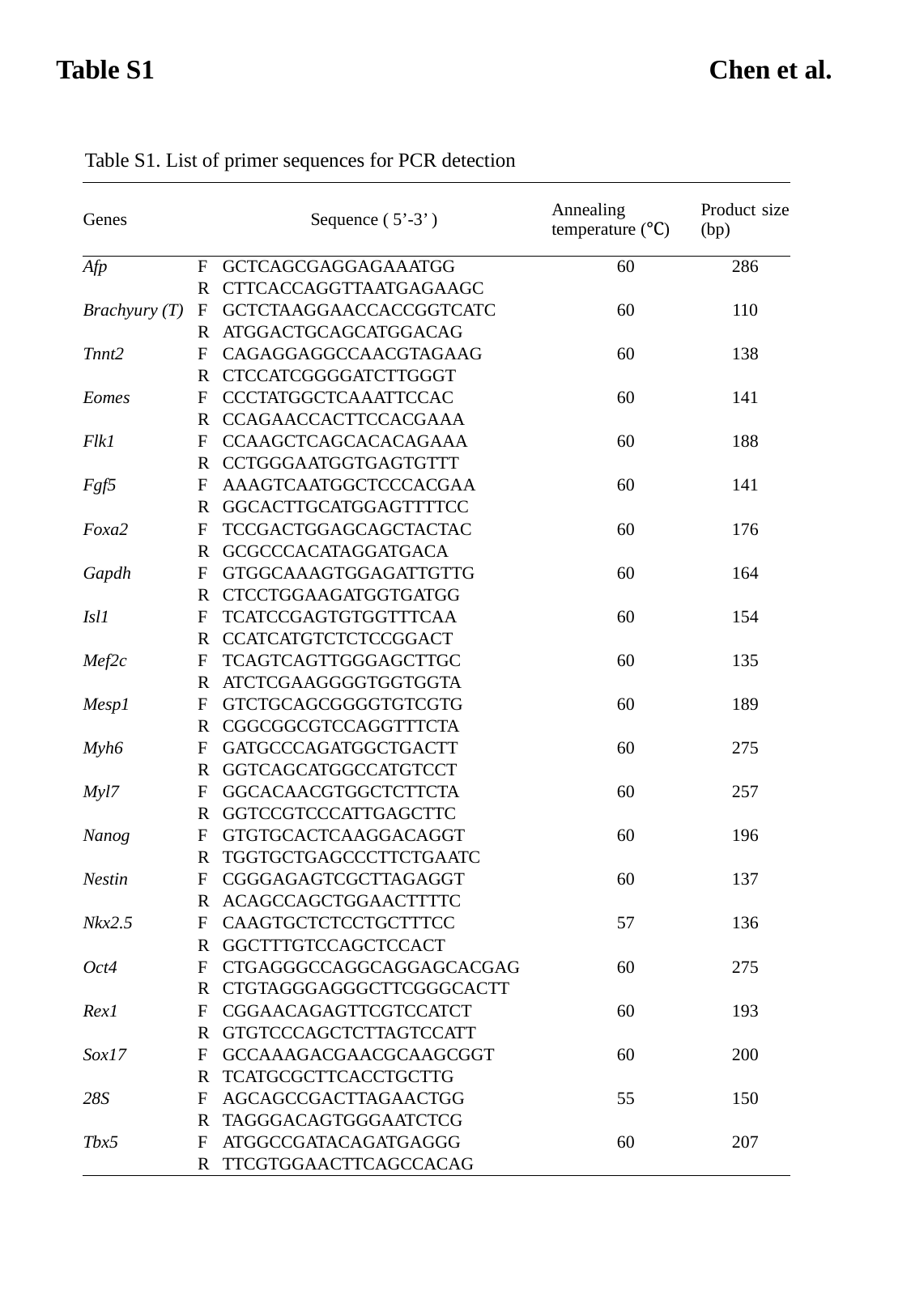

Table S1 Chen et al.
Table S1. List of primer sequences for PCR detection
| Genes | Sequence ( 5’-3’ ) | | Annealing temperature (℃) | Product size (bp) |
| --- | --- | --- | --- | --- |
| Afp | F | GCTCAGCGAGGAGAAATGG | 60 | 286 |
| | R | CTTCACCAGGTTAATGAGAAGC | | |
| Brachyury (T) | F | GCTCTAAGGAACCACCGGTCATC | 60 | 110 |
| | R | ATGGACTGCAGCATGGACAG | | |
| Tnnt2 | F | CAGAGGAGGCCAACGTAGAAG | 60 | 138 |
| | R | CTCCATCGGGGATCTTGGGT | | |
| Eomes | F | CCCTATGGCTCAAATTCCAC | 60 | 141 |
| | R | CCAGAACCACTTCCACGAAA | | |
| Flk1 | F | CCAAGCTCAGCACACAGAAA | 60 | 188 |
| | R | CCTGGGAATGGTGAGTGTTT | | |
| Fgf5 | F | AAAGTCAATGGCTCCCACGAA | 60 | 141 |
| | R | GGCACTTGCATGGAGTTTTCC | | |
| Foxa2 | F | TCCGACTGGAGCAGCTACTAC | 60 | 176 |
| | R | GCGCCCACATAGGATGACA | | |
| Gapdh | F | GTGGCAAAGTGGAGATTGTTG | 60 | 164 |
| | R | CTCCTGGAAGATGGTGATGG | | |
| Isl1 | F | TCATCCGAGTGTGGTTTCAA | 60 | 154 |
| | R | CCATCATGTCTCTCCGGACT | | |
| Mef2c | F | TCAGTCAGTTGGGAGCTTGC | 60 | 135 |
| | R | ATCTCGAAGGGGTGGTGGTA | | |
| Mesp1 | F | GTCTGCAGCGGGGTGTCGTG | 60 | 189 |
| | R | CGGCGGCGTCCAGGTTTCTA | | |
| Myh6 | F | GATGCCCAGATGGCTGACTT | 60 | 275 |
| | R | GGTCAGCATGGCCATGTCCT | | |
| Myl7 | F | GGCACAACGTGGCTCTTCTA | 60 | 257 |
| | R | GGTCCGTCCCATTGAGCTTC | | |
| Nanog | F | GTGTGCACTCAAGGACAGGT | 60 | 196 |
| | R | TGGTGCTGAGCCCTTCTGAATC | | |
| Nestin | F | CGGGAGAGTCGCTTAGAGGT | 60 | 137 |
| | R | ACAGCCAGCTGGAACTTTTC | | |
| Nkx2.5 | F | CAAGTGCTCTCCTGCTTTCC | 57 | 136 |
| | R | GGCTTTGTCCAGCTCCACT | | |
| Oct4 | F | CTGAGGGCCAGGCAGGAGCACGAG | 60 | 275 |
| | R | CTGTAGGGAGGGCTTCGGGCACTT | | |
| Rex1 | F | CGGAACAGAGTTCGTCCATCT | 60 | 193 |
| | R | GTGTCCCAGCTCTTAGTCCATT | | |
| Sox17 | F | GCCAAAGACGAACGCAAGCGGT | 60 | 200 |
| | R | TCATGCGCTTCACCTGCTTG | | |
| 28S | F | AGCAGCCGACTTAGAACTGG | 55 | 150 |
| | R | TAGGGACAGTGGGAATCTCG | | |
| Tbx5 | F | ATGGCCGATACAGATGAGGG | 60 | 207 |
| | R | TTCGTGGAACTTCAGCCACAG | | |

## Slide 2
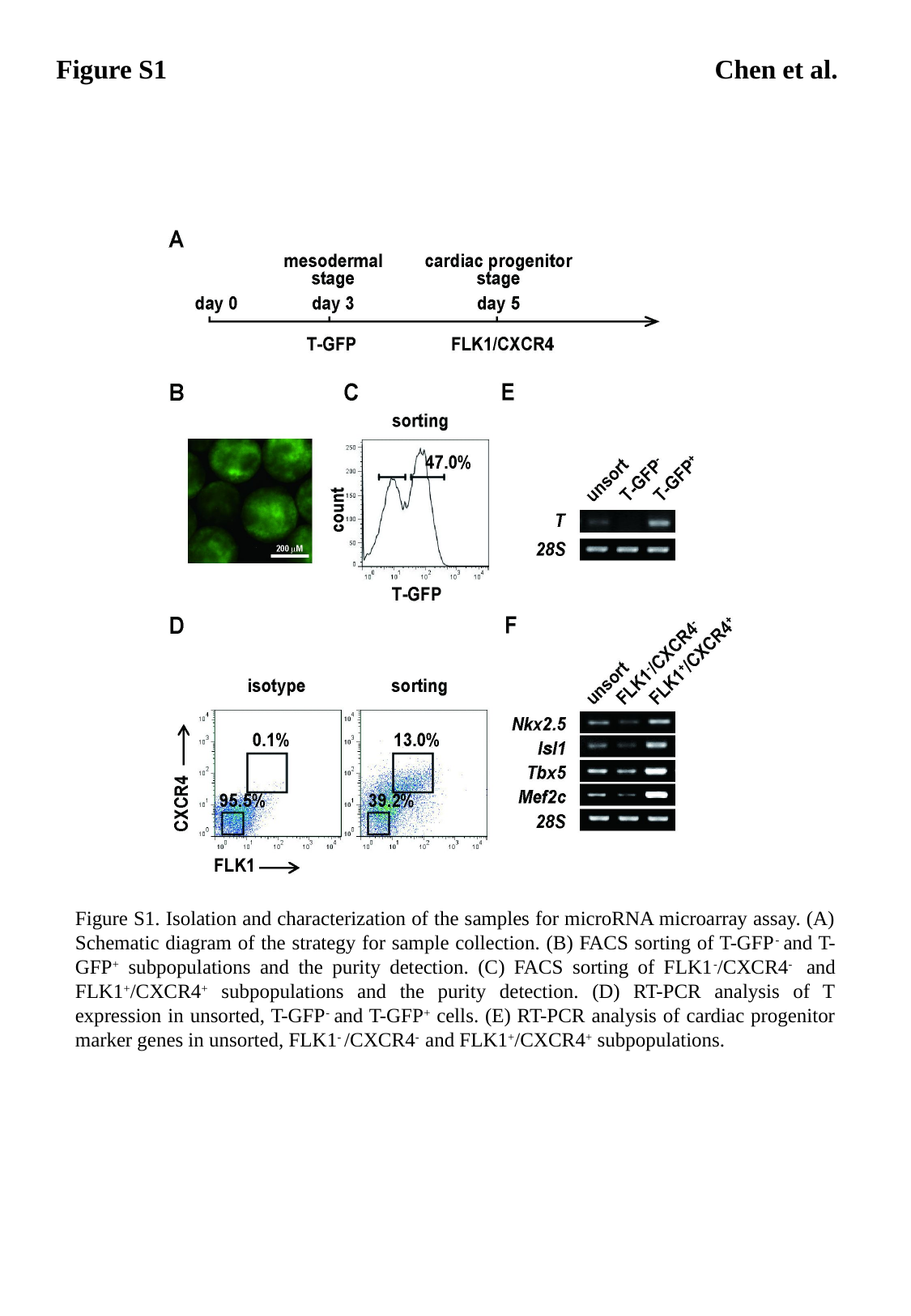

Figure S1 Chen et al.
Figure S1. Isolation and characterization of the samples for microRNA microarray assay. (A) Schematic diagram of the strategy for sample collection. (B) FACS sorting of T-GFP- and T-GFP+ subpopulations and the purity detection. (C) FACS sorting of FLK1-/CXCR4- and FLK1+/CXCR4+ subpopulations and the purity detection. (D) RT-PCR analysis of T expression in unsorted, T-GFP- and T-GFP+ cells. (E) RT-PCR analysis of cardiac progenitor marker genes in unsorted, FLK1- /CXCR4- and FLK1+/CXCR4+ subpopulations.

## Slide 3
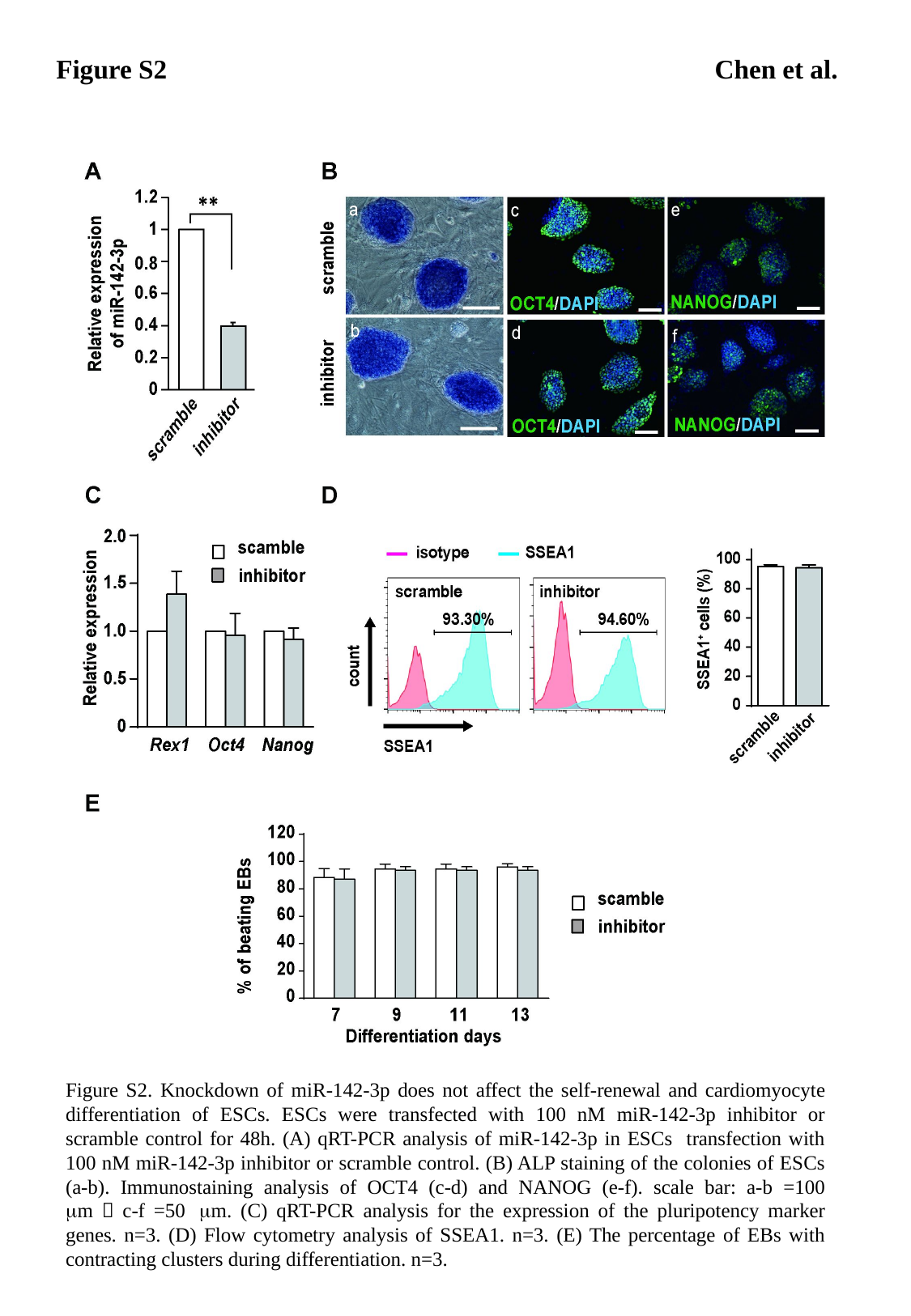

Figure S2 Chen et al.
Figure S2. Knockdown of miR-142-3p does not affect the self-renewal and cardiomyocyte differentiation of ESCs. ESCs were transfected with 100 nM miR-142-3p inhibitor or scramble control for 48h. (A) qRT-PCR analysis of miR-142-3p in ESCs transfection with 100 nM miR-142-3p inhibitor or scramble control. (B) ALP staining of the colonies of ESCs (a-b). Immunostaining analysis of OCT4 (c-d) and NANOG (e-f). scale bar: a-b =100 mm，c-f =50 mm. (C) qRT-PCR analysis for the expression of the pluripotency marker genes. n=3. (D) Flow cytometry analysis of SSEA1. n=3. (E) The percentage of EBs with contracting clusters during differentiation. n=3.

## Slide 4
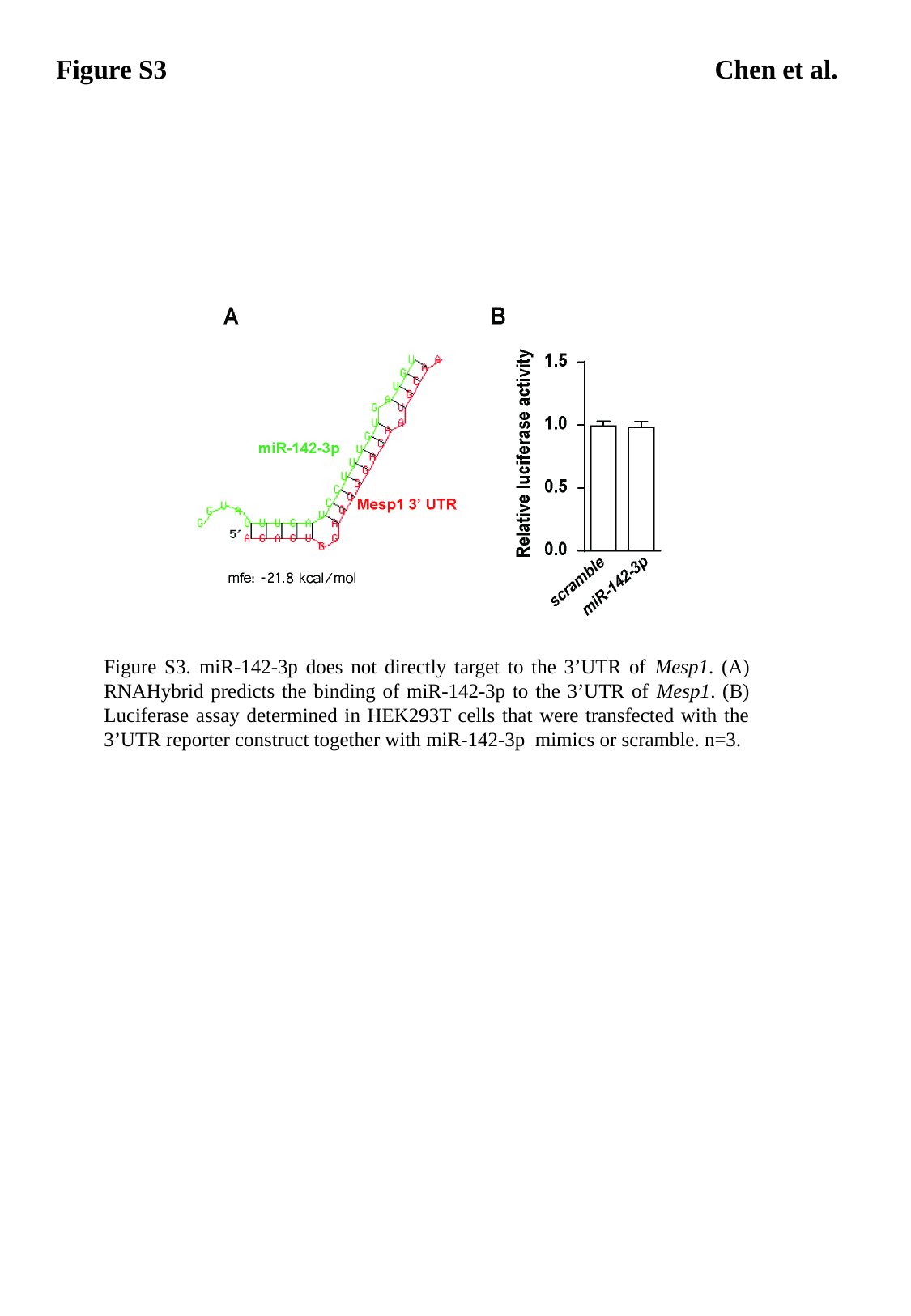

Figure S3 Chen et al.
Figure S3. miR-142-3p does not directly target to the 3’UTR of Mesp1. (A) RNAHybrid predicts the binding of miR-142-3p to the 3’UTR of Mesp1. (B) Luciferase assay determined in HEK293T cells that were transfected with the 3’UTR reporter construct together with miR-142-3p mimics or scramble. n=3.
